# Testing the portal imager GLAaS algorithm for machine quality assurance

**DOI:** 10.1186/1748-717X-3-14

**Published:** 2008-05-21

**Authors:** G Nicolini, E Vanetti, A Clivio, A Fogliata, G Boka, L Cozzi

**Affiliations:** 1 Oncology Institute of Southern Switzerland, Medical Physics Unit, Bellinzona, Switzerland; 2 University of Lausanne, Faculty of Medicine, Lausanne, Switzerland; 3 University of Milan, Medical Physics Specialisation School, Milan, Italy; 4 Latvian Oncology Center of Riga Eastern University Clinical Hospital. Dept. of Dosimetry., Riga, Latvia

## Abstract

**Background:**

To report about enhancements introduced in the GLAaS calibration method to convert raw portal imaging images into absolute dose matrices and to report about application of GLAaS to routine radiation tests for linac quality assurance procedures programmes.

**Methods:**

Two characteristic effects limiting the general applicability of portal imaging based dosimetry are the over-flattening of images (eliminating the "horns" and "holes" in the beam profiles induced by the presence of flattening filters) and the excess of backscattered radiation originated by the detector robotic arm supports. These two effects were corrected for in the new version of GLAaS formalism and results are presented to prove the improvements for different beams, detectors and support arms. GLAaS was also tested for independence from dose rate (fundamental to measure dynamic wedges).

With the new corrections, it is possible to use GLAaS to perform standard tasks of linac quality assurance. Data were acquired to analyse open and wedged fields (mechanical and dynamic) in terms of output factors, MU/Gy, wedge factors, profile penumbrae, symmetry and homogeneity. In addition also 2D Gamma Evaluation was applied to measurement to expand the standard QA methods. GLAaS based data were compared against calculations on the treatment planning system (the Varian Eclipse) and against ion chamber measurements as consolidated benchmark. Measurements were performed mostly on 6 MV beams from Varian linacs. Detectors were the PV-as500/IAS2 and the PV-as1000/IAS3 equipped with either the robotic R- or Exact- arms.

**Results:**

Corrections for flattening filter and arm backscattering were successfully tested. Percentage difference between PV-GLAaS measurements and Eclipse calculations relative doses at the 80% of the field size, for square and rectangular fields larger than 5 × 5 cm^2 ^showed a maximum range variation of -1.4%, + 1.7% with a mean variation of <0.5%. For output factors, average percentage difference between GLAaS and Eclipse (or ion chamber) data was -0.4 ± 0.7 (-0.2 ± 0.4) respectively on square fields. Minimum, maximum and average percentage difference between GLAaS and Eclipse (or ion chamber) data in the flattened field region were: 0.1 ± 1.0, 0.7 ± 0.8, 0.1 ± 0.4 (1.0 ± 1.4, -0.3 ± 0.2, -0.1 ± 0.2) respectively. Similar minimal deviations were observed for flatness and symmetry.

For Dynamic wedges, percentage difference of MU/Gy between GLAaS and Eclipse (or ion chamber) was: -1.1 ± 1.6 (0.4 ± 0.7). Minimum, maximum and average percentage difference between GLAaS and Eclipse (or ion chamber) data in the flattened field region were: 0.4 ± 1.6, -1.5 ± 1.8, -0.1 ± 0.3 (-2.2 ± 2.3, 2.3 ± 1.2, 0.8 ± 0.3) respectively.

For mechanical wedges differences of transmission factors were <1.6% (Eclipse) and <1.1% (ion chamber) for all wedges. Minimum, maximum and average percentage difference between GLAaS and Eclipse (or ion chamber) data in the flattened field region were: -1.3 ± 0.7, -0.7 ± 0.7, -0.2 ± 0.2 (-0.8 ± 0.8, 0.7 ± 1.1, 0.2 ± 0.3) respectively.

**Conclusion:**

GLAaS includes now efficient methods to correct for missing "horns" and "holes" induced by flattening filter in the beam and to compensate for excessive backscattering from the support arm. These enhancements allowed to use GLAaS based dosimetric measurement to perform standard tasks of Linac quality assurance with reliable and consistent results. This fast method could be applied to routine practice being also fast in usage and because it allows the introduction of new analysis tools in routine QA by means, e.g., of the Gamma Index analysis.

## 1. Background

Electronic portal imagers based on amorphous silicon flat panels are widely available in clinics and of natural interest for dosimetric purposes due to their intrinsic features. Many efforts have been put in develop methods to use these detectors for pre-treatment IMRT verification because of the possibility to reduce dramatically the time needed to perform the quality assurance processes compared to other devices, normally too time consuming.

A lot of publications investigated the performances and characteristics of the amorphous silicon (aSi) detector response [1-8]. One of the key factors, for dosimetric purposes, of aSi detectors is certainly their linear response in dose and dose rate, feature that allows a theoretically simple calibration process and a direct usage as dosimeters in many clinical and physical applications. One limiting factor, that often blocked a wide dosimetric usage of aSi's is that, in most of the cases, these detectors are part of the electronic portal imaging systems attached to linear accelerators and, in order to produce better image quality on the patients, basic detector calibration includes corrections for dark current and flood field aiming to generate an over flattened image from open fields. The consequence is that there is a basic difficulty in reproducing the off-axis ratio of normal clinical beams generated by the flattening filter (and other components) and ''corrected" for by the imager electronics. To complicate the dosimetric usage of aSi detectors there is the need to properly determine their response (in terms of linearity slope) at different field sizes and different energies and spectra, e.g. for primary or transmitted radiation (through multileaf collimators or through wedges).

Another important fact that has been originally pointed out by [9,10] for the Varian Portal Vision but in principle relevant for all similar systems, relates to the fact that aSi detectors are mounted on support arms connected to linacs without sufficient back-scatter material (due to mechanical reasons) to avoid or minimize the influence of the arm itself in the signal generation (some radiation back-scattered by the arm impinges on the aSi active area). As a consequence of this fact, the group of Siebers measured up to 5% asymmetry in the detector signal when changing field size from the conditions of image calibration (the largest field size) due to the different amount of backscattered radiation.

In summary, the problems mentioned above, together with some other practical difficulty and the absence of integrated software tools, limited the usage of aSi detectors as standard dosimeters to perform basic quality assurance tasks in radiation oncology. To achieve this goal, various algorithms converting the raw data acquired by the imagers into dose readings have to be implemented and tuned to overcome the undesired features variably affecting the dose response.

Our group developed and implemented in clinical practice such an algorithm, called GLAaS [11,12] to convert images from the aSi detectors PV-aS500 and PV-aS1000 from Varian Medical Systems, into dose matrices. In the previous publications the application of GLAaS was discussed and reported limitedly to pre-treatment IMRT verifications. GLAaS is a calibration algorithm mainly based on the application, on a pixel-by-pixel basis, of specific dose response curve parameters, pre-determined in an empiric way, and accounting for field size, primary or transmitted radiation and dynamic movement of multileaf collimator (for IMRT); the calibration could be performed at any desired depth in water equivalent materials. GLAaS did not added any 'calculation' or 'convolution' element in the process as this would correspond, in practice, to generate a simplified dose calculation engine from measured data for comparison against other measurements or other calculation engines (e.g. from the treatment planning systems). Power of GLAaS is the capability, with a minimal data manipulation, namely a simple direct calibration process, to convert raw measurements into absolute dose matrices usable for a variety of applications. In addition, the methods developed for GLAaS are quite flexible since, most of the parameters needed for its application are either determined on a single shot basis during its ''commissioning" or are derived from information contained, e.g., in the DICOM-RT structures of RT-Plans if GLAaS is applied to verify measurements against calculations performed by treatment planning systems (TPS).

Aim of the present study is to report about recent improvements to the basic GLAaS to better account for the general weak points mentioned above: a correction method to take into account the variation of off-axis ratio mostly determined by the flattening filter (FF) and a correction for the different arm backscattering when different field sizes are applied. In addition, GLAaS has been validated and adapted to operate with different dose rates (either fixed or variable during data acquisition) testing the eventual problem of saturation at high frequencies (depending from the read-out electronics). Finally, GLAaS, have been validated also for high dose per field deliveries (so far it was used in clinical applications limited to 2 Gy per field).

The reason for exploring these improvements was the intention to generalize the field of application of GLAaS based dosimetry moving from IMRT specific tests to periodic Linac Quality Assurance programmes of the radiation beams. For this reason, data will be reported about investigations performed on a variety of test conditions on open square, rectangular, symmetric or asymmetric fields as well as for fields with mechanical or dynamic wedges. The results on output and wedge factors and on beam penumbra, homogeneity and symmetry characteristics will demonstrate the potentials of GLAaS as a fast and practical tool for routine periodic machine based quality assurance procedures. A further step, currently in its final development stage, will expand GLAaS to the verification of arc therapies, particularly for dynamic conformal arcs and intensity modulated arcs with fixed or variable beam delivery features (e.g. variable dose rate).

## 2. Methods

The GLAaS algorithm [11] to convert raw images acquired with the portal imager into dose matrices has been used for this study. GLAaS has been configured to convert images acquired without any buildup on the PV cassette into dose at the depth of maximum dose d_max _at the same source-detector distance SDD.

A detailed description of the GLAaS algorithm, developed originally for pre-treatment IMRT verification, can be found in the original manuscript [11] and the description of its extension to the set-up setting allowing converting raw images into dose matrices at the depth of d_max _is contained in [12]. In this study we adopted all the methods described there and the recommended measuring depth. A summary of the algorithm logic and of the main equations, as well as a review of the experimental set-up are provided here in Appendix 1.

As pointed out also in the appendix, given the different nature of the conventional radiation fields with respect to IMRT fields, the latter being built as sequences of variable MLC apertures, it was necessary to introduce some elementary change in the basic definitions of fields and segments (used to discriminate in the GLAaS between areas of detector receiving primary or transmitted radiation). For open and wedged fields, it was intuitively assumed that one single radiation segment is concurring to the image generation to which is applied the whole GLAaS computation for primary radiation (transmission below collimating jaws is assumed to be negligible). For dynamic wedges, in principle it should be necessary to define a sequence of segments of progressively smaller size, following the jaws during motion. In practice it is sufficient to use one single segment, defined by the largest jaws opening since this contribution dominates over the entire field delivery. More details are provided in appendix 1.

The present report is divided into two main sections: the first is mostly devoted to describe the improvements introduced in GLAaS concerning the limitations described in the introduction (and called here flattening filter and arm backscattering corrections). Also the verification of GLAaS sensitiveness to various dose rates is addressed. These improvements were necessary to expand GLAaS field of application to quality assurance procedures different from IMRT. The second part of the study is devoted to a summary of GLAaS performances when it is applied to radiation tests in the framework of routine linac Quality Assurance.

To perform the present study most of the data were acquired on a Clinac 6EX (6 MV beam) equipped with a Portal Vision PV-aS500/IAS2 (connected to the linac gantry through the robotic arm called R-arm). These data were used to test GLAaS improvements, machine QA of both static and dynamic (as dynamic wedges) fields. The following PV-aS500 parameters' setting was used: SyncMode = 0, Rows per PVSync = 384, Synchronized Delay = 0, Number of Reset Frames = 0.

To validate the generality of the improvements and to verify some of the features described below, some test were repeated also on a second linac with 6 and 18 MV photon beams and equipped with a PV-aS1000/IAS3 mounted on the so called Exact-arm. In this case, EPID parameter settings were: Acquisition Technique=Integrated Image, Readout=Sync-Integrated. Specific comments or results are here reported only if different from what is presented or useful for discussion.

For simplicity, unless explicitly mentioned, all results will refer to the 6MV beam. Results and findings for the high energy beam are fully consistent and would not add anything to the value of the report. In addition, the validation of the GLAaS performances on different beam energies was reported in [12] proving the independence of GLAaS from beam energy.

Most of the dose matrices used to validate the methods, were derived from calculations performed with the Varian Eclipse treatment planning system, version 7.5.51, using the AAA photon dose calculation algorithm, version 8.0.05. Eclipse calculations were performed in a water phantom, at the depth of the maximum dose, (d_max _= 1.5 cm for 6MV), at the distance SDD (source-detector distance) of 100 cm for PV-aS1000/IAS3 (or 140 cm for PV-aS500/IAS2).

To strengthen the validation process, also measurements performed with ion chambers were used as reference. These data were acquired in a real water phantom at the depth of d_max _and proper SDD with a 0.125 cm^3 ^volume ion chamber for point and profiles acquisitions. For Enhanced Dynamic Wedges (EDW) the linear array of 48 ion chamber PTW LA48, in the same measuring conditions, was used and in the tables and figures referenced simply as ion chamber.

### 2.1 Enhancing GLAaS

#### a) The flattening filter correction

The basic process of image calibration in an electronic portal imager, and in particular for the Varian PortalVision (PV), includes the acquisition of a field as wide as the detector area (a 'flood field') used to equalize the detector reading through the whole area to improve image quality. In this way, the effect of the flattening filter in the machine output, generating the well-known "horns" in the most peripheral region of the fields is mostly canceled from PV images. For dosimetry purposes, it is then necessary to re-include this feature of the radiation beams, eventually off-line, if the detector shall be used as a reliable dosimeter for open fields. For IMRT fields, as discussed in [12] this problem was of secondary importance since the significant contribution from radiation transmitted below the multileaf collimator smears out the effect. In this study we introduced a first order simple correction in GLAaS, on the primary radiation only, that operates through a simple correction matrix determined once during the configuration of the GLAaS and to be eventually updated if major interventions on beam steering are introduced. This matrix is obtained computing the ratio, point by point, between a reference dose matrix (measured or computed by Eclipse at the chosen configuration (in terms of source detector distance, SDD, and depth equal to d_max_)) for the field size covering the whole detector area, and the corresponding matrix from the imager where the points equal the dose on the central axis for that field. This correction matrix is used as a pixel by pixel multiplicative factor to be applied only to the primary radiation component in the GLAaS formalism. More sophisticated methods could be elaborated but the cost/effect benefit should be carefully evaluated with respect to this first-order elementary approach.

#### b) The PV arm backscattering correction

The backscatter radiation contribution originated by the portal imager support structure and discussed in [9,10], is automatically compensated by the flood field image acquisition during the imager calibration procedure and hence it is properly accounted for only for the largest field size covering the entire active detector area. When the field size is decreased, also the amount of backscattered radiation from the arm is decreased, and the intrinsic correction from the flood field tends to over-correct for this effect. Visually and quantitatively this ends, for smaller fields, in lowering the measured dose in the part of the field seeing the support arm, i.e. generating slightly asymmetric fields. The effect is more pronounced in the half portion of the beam toward the gantry, where the mechanical and electrical components of the arm are positioned. As for the flattening filter correction, the relevance of this effect on IMRT fields was of smaller importance compared to the proper management of the effective field size of the sliding window and to the proper discrimination between primary and transmitted radiation. For open and partially for wedged fields it is instead fundamental to minimise all systematic and known sources of perturbation in the measurements and, for this reason, a method to compensate for this effect was developed and implemented in GLAaS. Similarly to the flattening filter case, a first order correction method is used. For all the square fields acquired in the configuration phase (ranging from 5 × 5 cm^2 ^to the maximum allowed field size), the ratio between the readings of the half portion of the field image seeing the arm (in the Varian convention the +*y *direction) and the half portion of the field not seeing the arm (-*y*) was computed. These matrices were then made linear to obtain, per each matrix, a family of angular coefficients of the y profile bending as a function of x (the arm backscatter contribution is slightly not symmetric with respect to the x axis). Since the arm backscattering contribution depends quite strongly on the field size, being more intensive for smaller fields, the correction coefficients increases from large to small fields.

During GLAaS application to a generic field, a linear fit, as a function of the jaw aperture toward +*y*, is computed in order to select from the library the appropriate correction coefficients (slope of the bending) to be applied, pixel by pixel, at the primary radiation component of the formalism.

As for the previous method, this solution represents a pragmatic approach to solve a known and complex issue. Deep modeling of the arm back-scatter is in principle possible via, e.g., Monte Carlo simulations but this would require a detailed knowledge of the arm structures and a huge investment in terms of configuration.

#### c) Dose Rate independence

To explore the application of GLAaS to verification of dynamic wedges, it was necessary to validate the calibration procedure for all dose rates available on a linac (in our case: 100, 200, 300, 400, 500 and 600 MU/min) and to assess its desirable operational independence from it. The latter is fundamental for two reasons: i) it allows in commissioning phase, to configure GLAaS for only one dose rate only and *ii) *to use it during irradiations performed with variable dose rate as the dynamic wedges. The second objective could be reached also in the case of strong detector sensitivity from dose rate by using libraries of calibrations and appropriate interpolations but it would be obviously quite complex from the logic and practical point of view.

In addition, different read-out electronics (IAS2 and IAS3) are associated to the detectors and among various differences, one is potentially relevant at this stage. The amplitude of the readable signal (over the number of frames between different detector cleanings) is limited to 14 bits in IAS2 while it is virtually not limited for IAS3. This limitation has a potential direct impact on the maximum dose rate usable on IAS2 to avoid saturation, or on the contrary, on the operational conditions (namely the SDD) to be used with higher dose rates to avoid saturation. IAS3 is not affected by potential limitations in dose rate. For both systems (aS500/IAS2 and aS1000/IAS3) complete sets of calibrations were acquired for all available dose rates and GLAaS parameters have been recorded and compared.

Because of the limited read-out buffer mentioned above, for the IAS2 case, the experiments were carried out setting SDD at 140 cm, a distance sufficient to reduce with inverse square low, the signal impinging on the detector and allowing testing the operation under the nominal dose rates delivered from the linac. It has to be mentioned that the IAS2 electronic is, from the Varian point of view, an end-of-life product and, for future applications, only IAS3 electronics should be considered.

To test the practical independence from the dose rate used to calibrate GLAaS and the dose rate used to deliver a test field, various IMRT fields were acquired with different dose rates, and analysed with different GLAaS calibrations (performing all the permutations) and results will be summarised here. It is obvious that this validation has a fundamental implication on a longer perspective since GLAaS independence from dose rate would allow its application to any type of beam delivery with variable dose rate, particularly in the area of advanced intensity modulation (arc) therapies.

#### d) High dose per field

A complementary aspect of the enhancement process of GLAaS was the assessment of its usability for relatively high dose per field. In the IMRT framework, GLAaS was operated in a regime roughly ranging from 0 to 2 Gy per field (normally 0 to <1 Gy) while in principle, for generic quality assurance purposes, it could be necessary to expose the detectors to higher dose levels. To test this factor in a simple but comprehensive way (i.e. exploring a large dose variation within a single image acquisition), a set of IMRT fields were delivered and verified via GLAaS assigning different dose levels, ranging from 0.2 to 5 Gy to the maximum field dose. In principle, the possibility to use PV and GLAaS for any dose (even higher than 5 Gy) should be guaranteed by the fact that read-out electronics operates by averaging the signal of each pixel over a given number of frames (while the detector is reset without loosing any acquisition frame), and recording the corresponding readings together with the total number frames. With this operation mode, the detector channels do not saturate with increasing dose.

### 2.2 Exploring GLAaS for Machine Quality Assurance

The second part of the study was devoted to validate the usage of GLAaS for simple linac quality assurance radiation tests.

An intrinsic advantage of GLAaS is that it allows performing dosimetric analysis on truly 2D data with a spatial resolution of either ~0.4 mm (PV-aS1000) or ~0.7 mm (PV-aS500). On the contrary standard dosimetric tests for linac QA processes are based on measurements either "zero" dimensional (points) or mono-dimensional (series of points in a line like with array detectors) or, when bi-dimensional data are available as when using 2D matrix detectors, the spatial resolution is very coarse (from 5 to 10 mm in average) and/or the points in the matrices are arranged in pre-defined simple geometries as in the detectors used to perform star measurements or to analyse beam profiles in the main axis in few points. The intrinsic bi-dimensionality of GLAaS allows therefore investigating also evaluation methods based on several criteria, standard parameters or, e.g. the gamma analysis. In addition, it is possible to use, as reference for constancy checks any type of point measurements (if data like output factors or wedge factors are of interest) but also to use 2D measurements from other detectors or 2D calculations from treatment planning systems. In other words, GLAaS is compatible with a large variety of reference data to perform quality assurance tests. In the present work, given our previous experience in comparing GLAaS based measurements against treatment planning calculations, the standard reference was chosen to be the TPS but, whenever possible, results were benchmarked against measurements with ion chamber as described above.

#### a) Open fields

A set of 16 open fields, square and rectangular, has been selected, sizing from 3 × 3 to 30 × 30 cm^2^. Several parameters were recorded; in the present paper the following are reported:

*i*. Single point dose on the central axis (CAX): Output Factors (ratio between the readings of the test field and the 10 × 10 cm^2 ^field), and dose in Gy were compared between PV-GLAaS measurements, ion chamber measurements, and Eclipse calculations. GLAaS and Eclipse values were computed as the average over a square ROI ranging from 5 × 5 pixels for fields smaller than 3 × 3 cm^2 ^to 65 × 65 pixels (about 5 × 5 mm) for fields larger than 8 × 8 cm^2 ^centered on the CAX.

*ii*. Profiles on the main axes: penumbrae (distance between the 20% and the 80% dose level), minimum, maximum and average dose in the flattened region, defined as the central 80% of the field size were computed. A twofold comparison was conducted: PV-GLAaS against Eclipse calculations or against ion chamber measurements wherever available. The following summary results were reported:

- the percentage difference between the minimum dose from GLAaS and the minimum dose from Reference (Eclipse or ion chamber) in all points of the flattened region: R_min _= 100*(D_min_^GLAaS^-D_min_^Reference^)/D_min_^GLAaS^, where D_min _is the minimum dose value the flattened region.

- the percentage difference between the maximum dose from GLAaS and the maximum dose from Reference in all points of the flattened region: R_max _= 100*(D_max_^GLAaS^-D_max_^Reference^)/D_max_^GLAaS^, where D_max _is the maximum dose value in the flattened region.

- the percentage difference between the average dose from GLAaS and the average dose from Reference in all points of the flattened region: R_ave _= 100*(D_ave_^GLAaS^-D_ave_^Reference^)/D_ave_^GLAaS^, where D_ave _is the average dose value in the flattened region.

- the minimum value of the percentage difference, point by point, between GLAaS and Reference computed dose in the flattened region: min(100*(D^GLAaS^-D^Reference^)/D^GLAaS^). For this (and the following two) parameters only Eclipse was used as reference.

- the maximum value of the percentage difference, point by point, between GLAaS and Reference computed dose in the flattened region: max(100*(D^GLAaS^-D^Reference^)/D^GLAaS^).

- the average value of the percentage difference, point by point, between GLAaS and Reference computed dose in the flattened region: ave(100*(D^GLAaS^-D^Reference^)/D^GLAaS^).

R_min_, R_max_, R_ave _and all remaining results are reported as averages over all beams, both directions, all field sizes.

In addition, standard parameters used in routine QA analysis were computed and reported: the flatness, defined as [(D_max_-D_min_)/(D_max_+D_min_)] in percentage (IEC 60976), and the symmetry, defined as Maximum Dose Ratio in percentage: max [D(x)/D(-x)] (IEC 60976).

*iii*. 2-dimensional images (only for GLAaS and Eclipse doses): exploiting at maximum the potentialities of GLAaS, the Gamma Agreement Index (GAI), defined as the percentage of points inside the field size passing the gamma evaluation criteria [13] of DTA = 3 mm and ΔD = 2, 2.5, 3, and 3.5% was computed. The relatively large DTA threshold used for open fields permits to overcome possible criticalities in the penumbra region due to the different spatial resolutions (~0.4 or ~0.7 mm for the PV data, >1 mm for Eclipse). This gamma analysis is quite interesting and rather uncommon in normal QA practice and can generate new standards in the evaluation of periodic dosimetric controls.

To complement the overview of GLAaS performances on open fields that could be part of standard radiation tests, a set of 14 open asymmetric fields was acquired. These were defined as half or quarter beams with different field sizes (starting from the whole open 20 × 20 and 10 × 10 cm^2^). For those cases only output factors and profiles are recorded.

#### b) Enhanced Dynamic Wedges (EDW)

All EDW wedges (10, 15, 20, 25, 30, 45, and 60 degrees), IN and OUT directions (in the Varian systems, EDW are operated by the upper Y jaws and are defined IN or OUT if the Y1 or Y2 jaw is respectively moved during irradiation), for a 20 × 20 cm^2 ^field were acquired and compared with the corresponding Eclipse computations and LA48 measurements. Results are presented for Dose/MU, profiles (reporting minimum, maximum and average differences between computed and measured profiles) and 2D GAI (DTA = 3 mm, ΔD = 3%).

#### c) Mechanical wedge fields

All mechanical wedges (15, 30, 45, and 60 degree wedge), for a 10 × 10 cm^2 ^field were analysed in terms of Transmission Factors, wedge angles, profiles and 2D GAI (DTA = 3 mm, ΔD = 3%).

## 3. Results

### 3.1 Enhancing GLAaS

#### a) The flattening filter correction and the PV arm backscattering

**Figure 1:**
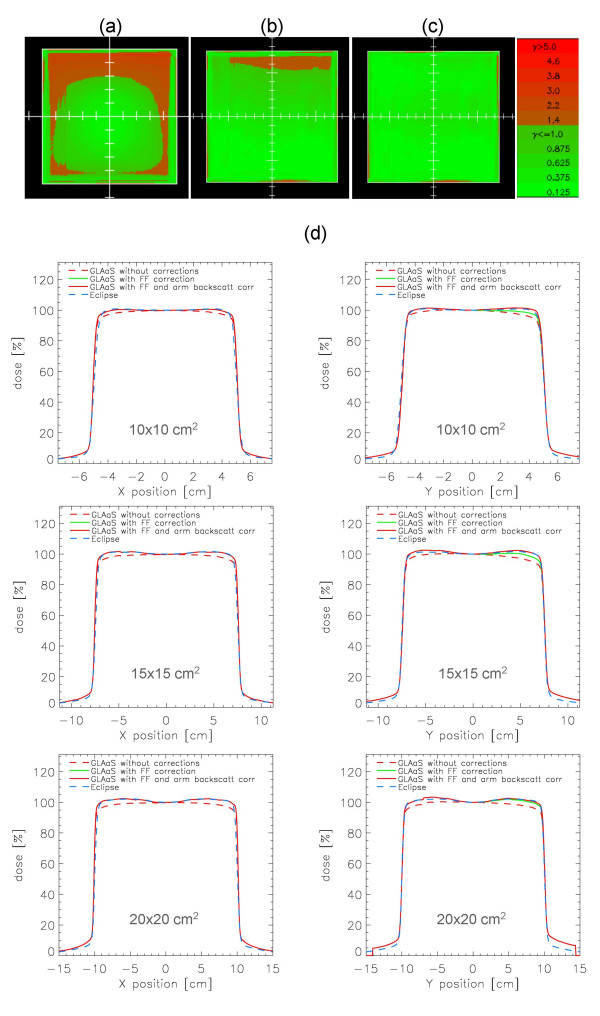
Flattening filter and arm-backscattering correction. Example for an open 15 × 15 cm^2 ^field of Gamma Evaluation matrices (DTA = 3 mm, ΔD = 3%) a) without corrections, b) with flattening filter correction, c) with both flattening filter and arm backscattering correction, d) profiles in *x *and *y *directions for 10 × 10, 15 × 15 and 20 × 20 cm^2 ^fields from Eclipse calculations, PV-GLAaS without, with flattening filter, and with flattening filter + arm backscattering corrections included. Data are shown at d_max _for a beam energy of 6 MV.

The application of the flattening filter and PV arm backscattering were tested for several field sizes. Examples are shown in figure 1 where the Gamma evaluation matrices (determined with DTA = 3 mm and ΔD = 3%) are presented for an open 15 × 15 cm^2 ^field without any correction, with flattening filter and with arm backscattering (R-arm in this example). The profiles shown in the figure for three different field sizes are normalized to 100% at the CAX in both *x *and *y *directions and show data from Eclipse calculations, PV-GLAaS measurements without or with the various corrections. Concerning arm backscattering, the effect is qualitatively the same for the R or the Exact-arms therefore only data for R-arm are shown for simplicity but results are equivalent in the other case.

From the profiles shown, it is easy to appraise the progressive improvement from the starting GLAaS data (flat profiles) to the presence of the expected 'hole' in the middle and 'horns' towards the edges to finally the compensation for the profile asymmetry in y. This pattern is not present in the GLAaS when not corrected for flattening filter, and it is on the contrary present in the corresponding not corrected gamma evaluation matrix, while the field inhomogeneity is better modeled when the flattening filter correction is accounted for (disappearing the 'horns' and 'hole' from the gamma evaluation matrix).

The difference between GLAaS and Eclipse dose for the corrected and uncorrected profiles at the level of the 80% of the field size (the edge of the flattened region) are reported in table 1, averaged over all open fields larger than 5 × 5 cm^2 ^analysed in the present study.

**Table 1:** Impact of flattening filter and arm backscatter corrections: percentage difference between PV-GLAaS measurements and Eclipse calculations relative doses at the 80% of the field size, for square and rectangular fields larger than 5 × 5 cm^2^; values are the average ± SD, and range; data for 6MV beam at d_max_.

	No correction [%]	Flattening filter correction [%]	Flatt.Filter + arm Backscatt. correction [%]
-*x *dir.	-1.9 ± 1.3 [-3.3, -0.1]	-0.2 ± 0.4 [-0.8, +0.3]	-0.2 ± 0.4 [-0.9, +0.3]
+*x *dir.	-1.9 ± 1.4 [-3.5, +0.1]	-0.2 ± 0.3 [-0.8, +0.1]	-0.2 ± 0.3 [-0.8, +0.0]
-*y *dir.	-1.7 ± 0.7 [-3.2, -0.5]	+0.6 ± 0.7 [-0.6, +1.8]	+0.5 ± 0.7 [-0.6, +1.7]
+*y *dir.	-4.2 ± 1.6 [-5.3, -0.3]	-1.8 ± 0.7 [-2.4, -0.3]	-0.2 ± 0.8 [-1.4, +1.2]

To retrospectively assess the impact of using this set of corrections, a representative set of IMRT pre-treatment verification analysed with the native GLAaS implementation, were reprocessed with the new enhanced release. With the inclusion of the flattening filter correction, the mean Gamma value of IMRT fields decreased of <10% from an average of 0.27 to 0.25 over the last 100 fields verified for clinical treatments while the Gamma Agreement Index improved only of few tenth of percentage. Similarly, the addition of the arm backscattering correction affected only in a minimal extent (mostly not visible) the IMRT pre-treatment results. These findings confirmed the original assumptions made in [11,12] that in IMRT, the complex pattern of delivery and the relevance of radiation transmitted below the MLC, masks strongly these features that are, on the contrary, important to be properly managed for open fields.

#### c) Dose Rate independence

The GLAaS configuration parameters derived from fit procedures according to the formalism shortly described in appendix, are summarized in table 2 for the two systems investigated. Data are reported as averages and standard deviations of the average of the calibrations parameters obtained from acquisitions at 100, 200, 300, 400, 500, and 600 MU/min. All fit parameters of the GLAaS formalism resulted equivalent within the measurement errors whichever the dose rate. This is a confirmation of the independence from dose rate of the detector response on one side and of the robustness of the GLAaS procedure that preserves this fundamental feature of aSi systems.

**Table 2:** GLAaS configuration parameters: average (± SD) values over all dose rates from 100 to 600 MU/Gy

Parameter	aS500/IAS2 Mean ± %SD	aS1000/IAS3 Mean ± %SD
*c *(eq. 2 Appendix 1)	1.197 ± 0.3%	1.255 ± 0.2%
*d *(eq. 2 Appendix 1)	-8.755 10^-2 ^± 1.6%	-1.121 10^-1 ^± 1.1%
*a *(eq. 3 Appendix 1)	-3.179 10^-6 ^± 1.9 %	-4.29 10^-6 ^± 1.6 %
*b *(eq. 3 Appendix 1)	1.456 10^-5 ^± 1.6%	1.993 10^-5 ^± 1.2%

**Figure 2:**
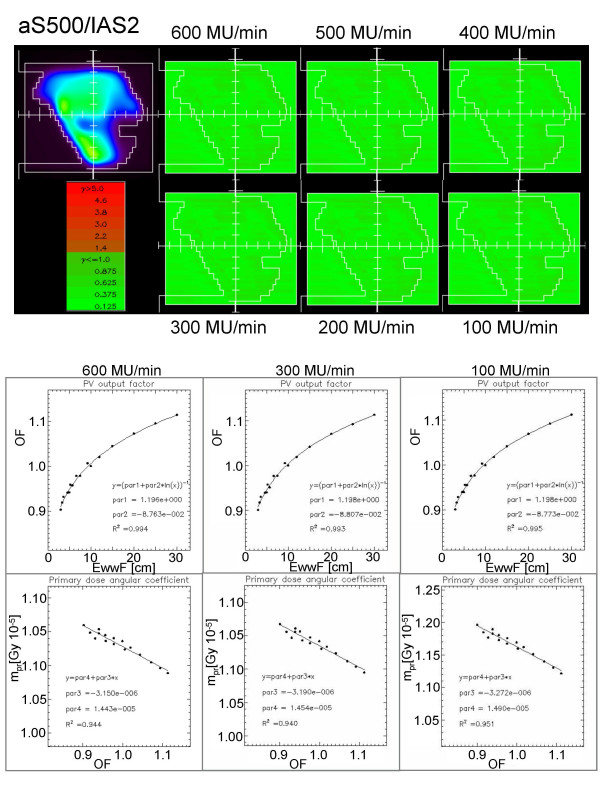
Summary of Dose Rate independence study for the IAS2 read-out electronics (associated to the PV-aS500 detector). Example of Gamma Evaluation matrices (DTA = 3 mm, ΔD = 3%) for a field measured with beam delivery operated at dose rates from 100 to 600 MU/min while dose calculation was performed at 300 MU/min. Plots of the calibration data acquired at different dose rates and corresponding fits.

**Figure 3:**
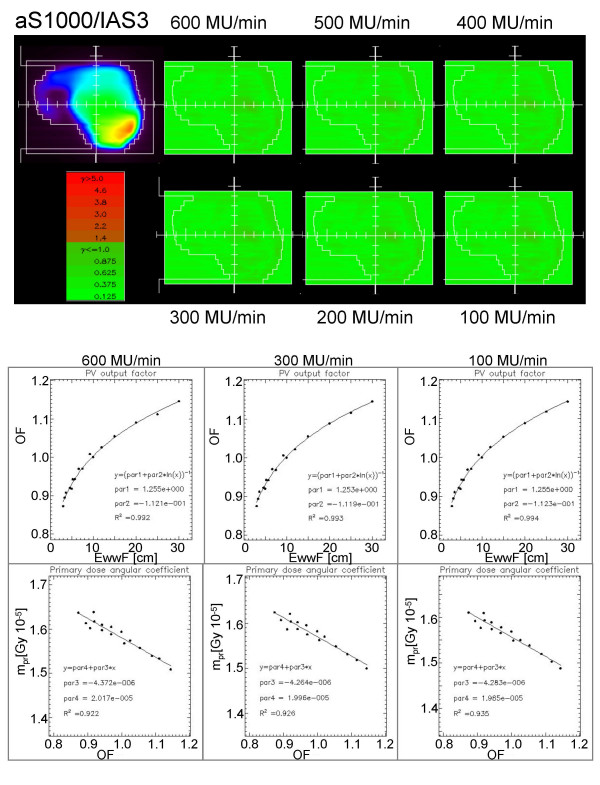
Summary of Dose Rate independence study for the IAS3 read-out electronics (associated to the PV-aS1000 detector). Example of Gamma Evaluation matrices (DTA = 3 mm, ΔD = 3%) for a field measured with beam delivery operated at dose rates from 100 to 600 MU/min while dose calculation was performed at 300 MU/min. Plots of the calibration data acquired at different dose rates and corresponding fits.

Validation tests were performed as described in the methods with several IMRT fields acquired with all dose rates and analysed mixing the conditions according to all permutations. In general, no difference was observed in the results (GAI, mean gamma and standard deviation) in all conditions confirming the possibility to perform only one GLAaS calibration and to use GLAaS with any dose rate. Figures 2 (aS500/IAS2) and 3 (aS1000/IAS3) present one example of IMRT field acquired with a given dose rate and reanalyzed with GLAaS parameters from all different dose rates; as mentioned, it is impossible to discriminate between the different gamma matrices and to identify the one from the proper matching of dose rates in acquisition and reprocessing. Figures 2 and 3 show also the results of the configuration process in terms of plots of experimental data and fit curves for output factors vs. effective window width and for angular coefficients vs. output factor according to the GLAaS formalism. These figures better substantiate the independence of the GLAaS formalism from the adopted dose rate.

#### d) High dose per field

The Gamma Agreement Index was computed for the set of several IMRT beams delivered with different dose per field. The independence of GLAaS from dose per field was verified through the assessment of the Gamma Agreement Index between each delivery and the corresponding calculation. The standard deviation of GAI over all tested cases resulted < 0.3% for an average GAI >99%. Mean and Standard deviation of GAI in normal clinical practice is 99.3 ± 0.9 [12]. This means that the observed variation due to different dose levels (from 0.2 to 5 Gy) is significantly smaller (one third) of the normal observed uncertainty and, therefore, GLAaS performances can be considered independent from this factor in a wide range of clinical doses. Prospectively, as for the dose rate study, this could also have relevant implications in the case of advanced IMRT techniques.

### 3.2 GLAaS for Machine QA

#### a) Open fields

Results on Output Factor and Dose/MU on the CAX are reported in table 3 as percentage difference between GLAaS and Eclipse, GLAaS and ion chamber, and, to benchmark findings, between Eclipse and ion chamber. Mean differences are limited within ± 1%. In particular, the smallest deviations are found in the comparison between GLAaS and ion chamber measurements, with a maximum variation of 1.1% over the whole set of measured fields, confirming the quality and robustness of GLAaS based dosimetry.

**Table 3:** Open fields: average (± SD) percentage difference between GLAaS and Reference (Eclipse or ion chamber) of output factors and Dose/MU over 16 analysed square and rectangular fields, and 14 asymmetric fields.

	GLAaS vs. Eclipse [%]	GLAaS vs. IonCh. [%]	Eclipse vs. IonCh. [%]
*Square and rectangular fields*			
Output factor:	-0.4 ± 0.7 [-1.3, +1.3]	-0.5 ± 0.4 [-1.1, 0.0]	-0.2 ± 0.4 [-1.3, +0.4]
Dose/MU:	0.8 ± 0.6 [-0.1, +2.3]	0.6 ± 0.3 [0.0, +1.1]	-0.2 ± 0.4 [-1.4, +0.3]
*Asymmetric fields*			
Output factor:	-0.8 ± 0.7 [-2.3, 0.0]	-	-
Dose/MU:	-0.3 ± 0.7 [-1.7, +0.6]	-	-

Profiles were differently analysed in the flattened region and at field edge. In this second case, the mean difference between the field penumbrae measured with GLAaS and computed by Eclipse for all open fields was investigated. A small overestimation of the penumbrae computed by Eclipse relatively to GLAaS measurements in the *y *direction was recorded (0.3 ± 0.4 mm, range [-0.2, +1.3] mm). The difference increased in the *x *direction: 1.4 ± 0.3 mm, [+1.0, +2.2]. This result was expected for two reasons. In Eclipse only profile data in one direction are used to configure the system (x profiles that are along the motion direction of the lower jaws in the gantry head) and the data used to commission Eclipse were measured with a resolution of 2.5 mm rather coarse if compared to the PV resolution of 0.784 mm (aS500) or 0.392 mm (aS1000). As a consequence, penumbrae from Eclipse are expected to be larger in *x *direction because of resolution while, concerning y, wider penumbrae are expected in GLAaS because this is the direction of motion of upper jaws, not perfectly modeled in Eclipse (here penumbrae in the two main directions are identical). In effect, penumbrae measured with GLAaS were about 1 mm wider in y compared to x.

Results of the differences between profiles in the flattened region are reported in table 4 for all fields larger than 5 × 5 cm^2^. In table 5, some of the standard parameters commonly used for profile analysis have been reported for GLAaS processed measured images, Eclipse calculations, and ion chamber measurements, for some field sizes, *x *and *y *directions.

**Table 4:** Open fields: minimum, maximum and average (± SD) percentage difference between GLAaS and Reference (Eclipse or ion chamber) for fields larger than 5 × 5 cm^2^, in the flattened region.

	GLAaS vs. Eclipse Mean ± SD [Range] [%]	GLAaS vs. IonCh. Mean ± SD [Range] [%]
R_min _= 100*(D_min_^GLAaS^-D_min_^Ref^)/D_min_^GLAaS^	0.1 ± 1.0 [-0.7, +3.9]	1.0 ± 1.4 [+0.1, +2.6]
R_max _= 100*(D_max_^GLAaS^-D_max_^Ref^)/D_max_^GLAaS^	0.7 ± 0.8 [-0.3, +2.4]	-0.3 ± 0.2 [-0.4, 0.0]
R_ave _= 100*(D_ave_^GLAaS^-D_ave_^Ref^)/D_ave_^GLAaS^	0.1 ± 0.4 [-0.5, +0.6]	-0.1 ± 0.2 [-0.3, +0.1]
min(100*(D^GLAaS^-D^Ref^)/D^GLAaS^)	1.1 ± 1.0 [0.0, 3.9]	-
max(100*(D^GLAaS^-D^Ref^)/D^GLAaS^)	-0.6 ± 0.5 [-1.5, 0.0]	-
ave(100*(D^GLAaS^-D^Ref^)/D^GLAaS^)	0.1 ± 0.4 [-0.5, 0.6]	-

**Table 5:** Open fields: summary of main parameters from profile analysis; data are shown for three exemplifying field sizes.

Field/axis		D_min _* [%]	D_max _* [%]	D_ave _* [%]	Flatness [%]	Symmetry [%]
5 × 5, *y*	Ion chamber	93.6	100.0	98.8	3.3	100.4
	Eclipse	93.9	100.1	99.6	3.2	100.0
	GLAaS	96.2	100.8	100.1	2.3	100.1
10 × 10, *y*	Ion chamber	98.8	100.8	100.2	1.0	100.5
	Eclipse	100.0	100.9	100.4	0.4	100.0
	GLAaS	100.0	101.5	100.8	0.8	100.0
20 × 20, *y*	Ion chamber	99.9	102.7	101.7	1.4	100.6
	Eclipse	100.0	102.2	101.4	1.1	100.1
	GLAaS	99.9	103.4	101.6	1.7	101.2
5 × 5, *x*	Ion chamber	94.7	100.3	99.0	2.9	100.4
	Eclipse	93.9	100.1	99.6	3.2	100.0
	GLAaS	97.7	100.1	99.4	1.2	100.0
10 × 10, *x*	Ion chamber	99.2	101.0	100.3	0.9	100.6
	Eclipse	100.0	100.9	100.4	0.4	100.1
	GLAaS	99.3	100.6	100.1	0.7	100.0
20 × 20, *x*	Ion chamber	99.7	102.6	101.4	1.4	100.4
	Eclipse	100.0	102.2	101.4	1.1	100.1
	GLAaS	99.7	102.5	101.3	1.4	100.2

* within flattened region

**Figure 4:**
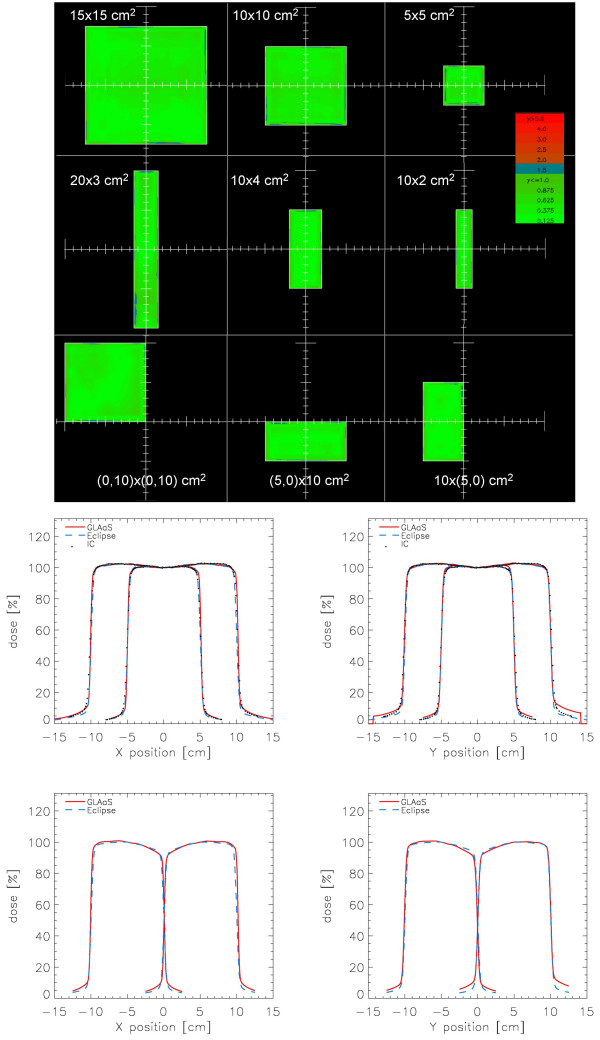
Examples of various verification (Gamma Evaluation matrices (DTA = 3 mm, ΔD = 3%) and profiles) for open square, rectangular and asymmetric fields. Profiles are shown in the *x *and *y *directions: first row: 10 × 10 and 20 × 20 cm^2 ^fields; second row: asymmetric (half beam) fields of 10 × 20 and 20 × 10 cm^2^. Data are reported for Eclipse calculations (blue dashed), GLAaS (red line), and ion chamber (black dots). Depth of measure or calculation was d_max _for a beam energy of 6MV.

The high quality of the agreement between Eclipse calculations and GLAaS measurements, can be appraised also in figure 4 where the gamma index maps for some field are shown as well as profiles in x and y for two square and two asymmetric fields. In the plots, Eclipse, GLAaS and ion chamber data are compared.

From the Gamma analysis of all fields, the average Gamma Agreement Index (DTA = 3 mm) computed over the whole field area for all square and asymmetric fields, resulted: 92.7 ± 6.0 %, 97.3 ± 1.3 %, 98.4 ± 0.9 %, and 99.2 ± 0.4 % for ΔD = 2, 2.5, 3, 3.5% respectively.

#### b) Enhanced Dynamic Wedges (EDW)

In table 6 Dose/MU on the CAX are reported as percentage difference between GLAaS and Eclipse, GLAaS and ion chamber measurements, and, as benchmark, between Eclipse and ion chamber. The best agreement was observed between GLAaS and ion chamber (maximum deviation smaller than 1.5%), while larger differences were found between GLAaS and Eclipse or between Eclipse and ion chamber. This, again, substantiates the role of GLAaS as a dosimeter equivalent to conventional ion chambers.

**Table 6:** EDW: average (± SD) percentage difference of Dose/MU between GLAaS and Reference (Eclipse or ion chamber), and range over 14 analysed EDW fields.

	GLAaS vs Eclipse [%]	GLAaS vs IonCh. [%]	Eclipse vs IonCh. [%]
Dose/MU:	-1.1 ± 1.6 [-4.8, +1.2]	0.4 ± 0.7 [-0.9, +1.4]	1.6 ± 1.3 [+0.1, +4.2]

**Figure 5:**
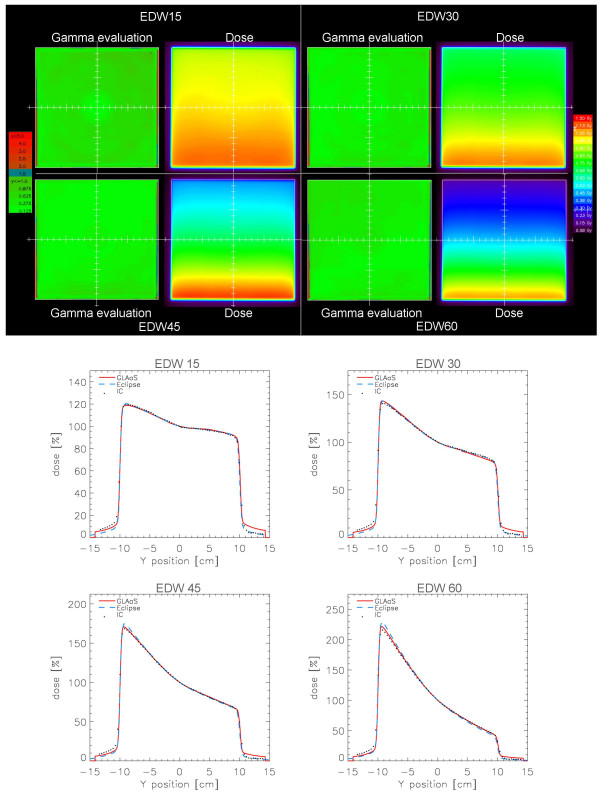
Examples of EDW verification showing Gamma Evaluation matrices (DTA = 3 mm, ΔD = 3%) and profiles in the *y *direction for 15, 30, 45, and 60° EDW ''IN" direction on a 20 × 20 cm^2 ^field. Data are reported for Eclipse calculations (blue dashed), GLAaS (red line), and ion chamber (black dots).

Table 7 summarises results of the differences between Eclipse and GLAaS profiles in the flattened region for all EDW fields. In the first part of the table results from all the wedge angles are reported while in the second part the 60 degree EDW was removed. The difference between the results of the two parts shows, as usual, that the 60 degree EDW presents the least accuracy in dose computation. The agreement between Eclipse and GLAaS, in average it is within 1.3% (1.7% if also EDW 60 is accounted for). Figure 5 shows GLAaS dose matrices and 2D gamma maps for 15, 30, 45 and 60 degrees EDW fields with also corresponding profiles in the wedge direction for GLAaS measurements, Eclipse calculations and for ion chamber measures (with LA48).

**Table 7:** EDW: minimum, maximum and average (± SD) percentage difference between GLAaS and Reference (Eclipse or ion chamber) in the flattened region.

	GLAas vs Eclipse Mean ± SD [Range] [%]	GLAaS vs IonCh. Mean ± SD [Range] [%]
*Angles 10 to 60 degree*		
R_min _= 100*(D_min_^GLAaS^-D_min_^Ref^)/D_min_^GLAaS^	0.4 ± 1.6 [-2.0, +4.4]	-2.2 ± 2.3 [-5.5, -0.3]
R_max _= 100*(D_max_^GLAaS^-D_max_^Ref^)/D_max_^GLAaS^	-1.5 ± 1.3 [-4.7, +0.5]	2.3 ± 1.2 [+1.0, +3.9]
R_ave _= 100*(D_ave_^GLAaS^-D_ave_^Ref^)/D_ave_^GLAaS^	-0.1 ± 0.3 [-0.9, +0.1]	0.8 ± 0.3 [+0.5, +1.1]
min(100*(D^GLAaS^-D^Ref^)/D^GLAaS^)	1.2 ± 1.2 [+0.5, +4.4]	-
max(100*(D^GLAaS^-D^Ref^)/D^GLAaS^)	-1.7 ± 1.1 [-4.7, -0.5]	-
ave(100*(D^GLAaS^-D^Ref^)/D^GLAaS^)	0.1 ± 0.2 [-0.2, +0.6]	-
*Angles 10 to 45 degree*		
R_min _= 100*(D_min_^GLAaS^-D_min_^Ref^)/D_min_^GLAaS^	-0.2 ± 0.7 [-2.0, +0.9]	-1.1 ± 0.8 [-2.0, -0.3]
R_max _= 100*(D_max_^GLAaS^-D_max_^Ref^)/D_max_^GLAaS^	-1.1 ± 0.9 [-3.2, +0.5]	1.8 ± 0.7 [+1.0, +2.5]
R_ave _= 100*(D_ave_^GLAaS^-D_ave_^Ref^)/D_ave_^GLAaS^	0.0 ± 0.2 [-0.4, +0.1]	0.6 ± 0.2 [+0.5, +0.8]
min(100*(D^GLAaS^-D^Ref^)/D^GLAaS^)	0.8 ± 0.3 [+0.5, +1.4]	-
max(100*(D^GLAaS^-D^Ref^)/D^GLAaS^)	-1.3 ± 0.8 [-3.2, -0.5]	-
ave(100*(D^GLAaS^-D^Ref^)/D^GLAaS^)	0.0 ± 0.1 [-0.2, +0.2]	-

As for the case of simple open fields, the good agreement of results is confirmed by the average Gamma Agreement Index computed over the whole field area between GLAaS and Eclipse dose matrices: 97.5 ± 1.1% (minimum value 95.4%) for DTA = 3 mm and ΔD = 3%.

#### c) Mechanical wedge fields

In table 8 the Transmission Factors (on the CAX for a 10 × 10 cm^2 ^field) are reported as percentage difference between GLAaS and Eclipse, GLAaS and ion chamber measurements, and, as benchmark, between Eclipse and ion chamber. The agreement between GLAaS and ion chamber is within 1.1%, better than with respect to Eclipse as in for the case of EDW.

**Table 8:** Mechanical wedges: average (± SD) percentage difference of Transmission Factors between GLAaS and Reference (Eclipse or ion chamber) over the 4 wedge insert directions.

	GLAaS vs Eclipse [%]	GLAaS vs IonCh. [%]	Eclipse vs IonCh. [%]
W1 - 15°	-1.3 ± 0.2	-1.1 ± 0.1	0.2 ± 0.1
W2 - 30°	-1.6 ± 0.1	0.2 ± 0.1	1.7 ± 0.1
W3 - 45°	-1.6 ± 0.1	-0.8 ± 0.1	0.8 ± 0.0
W4 - 60°	-1.3 ± 0.2	-0.8 ± 0.2	0.4 ± 0.0

Profile slopes are reported in table 9. Slopes were defined as 2*Artg(ΔDose/ΔDist), with ΔDose and ΔDist evaluated at the points located at half distance on the right and left sides respect to the field center. Results of the differences between Eclipse and GLAaS measured profiles in the flattened region are reported in table 10 for all analysed fields (all wedges, 10 × 10 and 20 × 20 cm^2 ^fields).

**Table 9:** Mechanical wedges: reconstructed profile slopes.

	GLAaS [°]	IonChamber [°]	Eclipse [°]
W1 - 15°	15.0	14.7	14.7
W2 - 30°	30.9	31.2	29.8
W3 - 45°	45.1	44.0	44.4
W4 - 60°	75.6	73.9	74.3

**Table 10:** Mechanical wedges: minimum, maximum and average (± SD) percentage difference between GLAaS and Reference (Eclipse or ion chamber) in the flattened region.

	GLAaS vs Eclipse Mean ± SD [Range] [%]	GLAaS vs Ion Chamber Mean ± SD [Range] [%]
R_min _= 100*(D_min_^GLAaS^-D_min_^Ref^)/D_min_^GLAaS^	-1.3 ± 0.7 [-2.0, -0.2]	-0.8 ± 0.8 [-1.8, +0.3]
R_max _= 100*(D_max_^GLAaS^-D_max_^Ref^)/D_max_^GLAaS^	-0.7 ± 0.7 [-1.5, +0.5]	0.7 ± 1.1 [-0.6, +2.8]
R_ave _= 100*(D_ave_^GLAaS^-D_ave_^Ref^)/D_ave_^GLAaS^	-0.2 ± 0.2 [-0.4, +0.2]	0.2 ± 0.3 [-0.1, +0.7]
min(100*(D^GLAaS^-D^Ref^)/D^GLAaS^)	0.3 ± 0.2 [0.0, +0.6]	-
max(100*(D^GLAaS^-D^Ref^)/D^GLAaS^)	-1.4 ± 0.8 [-2.4, -0.3]	-
ave(100*(D^GLAaS^-D^Ref^)/D^GLAaS^)	-0.2 ± 0.2 [-0.5, +0.1]	-

**Figure 6:**
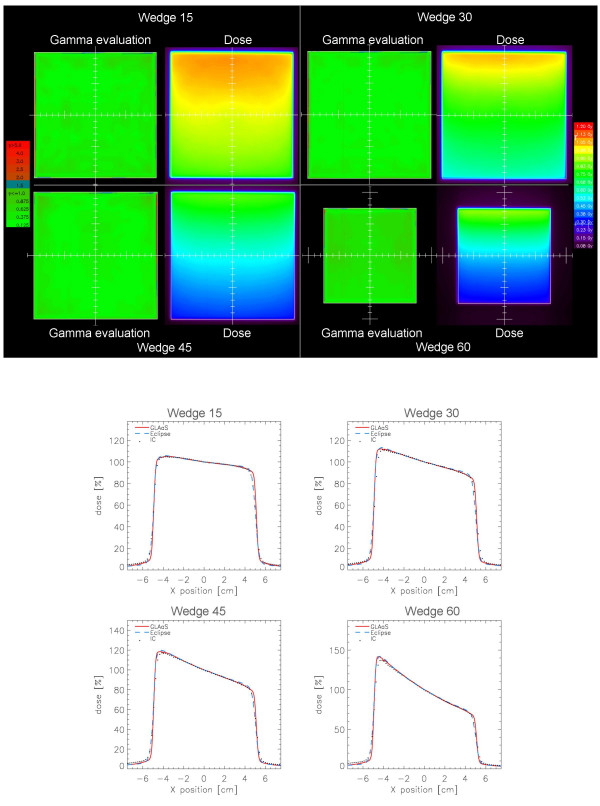
Examples of mechanical wedges verification showing Gamma Evaluation matrices (DTA = 3 mm, ΔD = 3%) and profiles in the wedge direction for 15, 30, 45, and 60° wedges on a 10 × 10 cm^2 ^field. Data are reported for Eclipse calculation (blue dashed), GLAaS (red line), and ion chamber (black dots).

Also in this case, figure 6 shows some examples of GLAaS dose matrices, 2D gamma maps and profiles for GLAaS, Eclipse and ion chamber data. The average Gamma Agreement Index computed over the whole field area between GLAaS and Eclipse dose matrices was: 98.7 ± 0.6% (minimum value 97.8%) for DTA = 3 mm and ΔD = 3%.

## 4. Discussion and Conclusion

The present report addressed some improvements introduced into the formalism of the GLAaS algorithm used to calibrate amorphous silicon based electronic portal imagers to convert raw images from into dose matrices at the depth of maximum dose. This enhanced version of GLAaS was motivated by the intention to apply GLAaS dosimetry to standard procedures of Quality Assurance of linac beams. In particular, GLAaS could be adopted to perform simple periodic stability control as determination of output and wedge factors or to monitor beam profile characteristics. In addition, from 2D dose matrices with high spatial resolution, it is possible to use GLAaS also to compute gamma index maps over the entire field areas, in the penumbra region and outside beams introducing new methods in routine quality assurance processes. A third point of interest is that GLAaS can be used using as reference data any type of measurement (including GLAaS itself) but can be operated also using as references calculations from planning systems. This could open also the possibility to perform extensive quality control to planning systems themselves or to verify linac stability over time against the data used to prepare treatments of patients.

The improvements introduced in this study were required to properly account for the 'holes' and 'horns' typical shape of radiation fields generated by the flattening filter and to compensate for the extra contribution to signals originated by backscattered radiation from the imager support arm. These are known issues in portal dosimetry, addressed also by other authors and the solution proposed in our study is, as GLAaS, empirical and pragmatic. Simple correction matrices (or coefficients), easily determined at the time of configuration for any type of detector or read-out electronic and beam quality.

To solve the backscattering problem also alternative solutions were proposed as result of the deep investigations of the Richmond group [9] that in 2005 [10] suggested, from Monte Carlo calculations, to add a lead layer to absorb the scattered radiation. More advanced modeling of the mentioned effects is possible but the quality of the results shown in this report allows confidence in the robustness of the simple method proposed.

Without the flattening filter and arm backscattering corrections, GLAaS was and is reliably usable for IMRT pre-treatment verification as shown by results in [11,12] but for wider application like generic quality assurance of linac beams (or planning systems) these were considered to be mandatory. This derives from the fact that both corrections are quantitatively relevant on the primary radiation component of the dosimetric signal and for open (or wedged) fields this is the completely dominant fraction. The case of IMRT is different because in this modality, radiation transmitted through the MLC cannot be ignored and play a significant role in the field modulation.

As mentioned repeatedly and systematically done in the study, given its bi-dimensional nature, it is obvious its compare GLAaS dosimetric data against dose calculations from the treatment planning systems; in our case the Varian Eclipse. It shall be nevertheless mentioned that this approach is reliable and safe only when computation is performed with sufficiently accurate algorithms. For the present study, the Anisotropic Analytical Algorithm AAA for photon dose calculation was applied, an algorithm deeply tested and presenting good agreement with ion chamber measurements in water for different settings [14]. Still, as shown by the present data, GLAaS allowed detecting some known features of the Eclipse-AAA system in terms of MU calculations and or penumbra evaluation proving its robustness and high reliability as an investigational tool. Its bi-dimensionality and the concomitant application of gamma index analysis suggests also that GLAaS dosimetry could be in this respect more informative than simple ion-chamber based investigations because it would allow the exploration of positive or negative beam features over the entire field area (and outside). In this respect GLAaS dosimetry is also superior to other 2D methods based on different commercial devices that are normally characterized by poor spatial resolution (ranging from 5 to 10 mm in general) and limited number of detection points (small areas or only privileged directions can be measured with decent spatial resolutions). In addition, time and easiness of execution is also an important factor. For example films have a long procedure of developing, scanning, conversion into dose through calibration curves depending on many factors (developer, energy,...) while linear arrays or 2D detectors are sometimes difficult to mount, and expensive.

Of final interest, there is also the observation that, when directly compared, GLAaS results and ion-chamber measurements showed the best agreement than the other combinations as a definitive prove of GLAaS proper implementation and value and the legitimacy to use GLAaS for routine tests, even in the framework of mandatory and legally binding procedures. GLAaS gives the possibility to check not only relative dose distributions, but also absolute dose values for any type of field, contrarily to what suggested e.g. by Budgell et al [15], where the EPID Quality Assurance was intended to check only the constancy of the parameters during time, despite of the specific value.

In summary, all the results shown, indicate that, a Quality Assurance program can reliably incorporate GLAaS dosimetry as an instrument for radiation tests to either monitor beam stability or to perform planning system validations (e.g. when new releases are issued). With GLAaS parameters like output factors, dose/MU, profiles, profile related parameters like flatness, symmetry, homogeneity and penumbrae can be quickly and reliably measured in good setting conditions of distance and depth (dmax is not the only option for GLAaS), and not only as reproducibility values. In addition, not addressed here but obvious also from previous publications, GLAaS dosimetry can be operated at any gantry angle and therefore it is a suitable and ideal method to solve some tricky issue of periodic quality assurance procedures.

A dedicated interface was developed for the purpose and is going to allow, in our institute, to perform routine activities with proper automatic calculation of all needed quality assurance parameters and immediate electronic recording as well as a variety of graphical interactive tools to perform users defined additional analysis.

Even if GLAaS appears to be extremely solid, it will not replace ion chamber measurements (e.g. depth dose measurements) but gives the possibility to enforce in critical areas, frequent beam checks being a fast and economic approach of beam testing.

PV-GLAaS has been demonstrated to be a comprehensive tool for QA in terms of pre-treatment IMRT verification [11,12], as well as for QA in terms of periodic beam check for any kind of fields: open, symmetric or asymmetric, EDW, wedge. MLC verification was not included in the present report and will be subject of a specific investigation to incorporate into GLAaS the possibility to analyse all characteristics, static and dynamic of MLC beams as well as dosimetric features of MLC systems.

Further ongoing studies, subject of future reports are focused on one extremely actual and important issue: the usage of GLAaS on advanced IMRT delivery methods, like (volumetric) modulated dynamic arc therapy. These investigations will be possible also given the proof, shown in the present study, of GLAaS independence from variable dose rate and from variable and high dose per field.

## Competing interests

The authors declare that they have no competing interests.

Dr. Luca Cozzi acts as Scientific consultant to Varian Medical Systems AG

## Authors' contributions

AF, GN and LC designed the study.

AF and LC wrote the manuscript

EV, AC, GN and GB performed data acquisition and processing.

AF, GN, LC, EV and AC developed the algorithms

EV and AC wrote the computer programmes

All authors reviewed and approved the manuscript

## Appendix 1

The GLAaS formalism was defined in detail in [11,12]. Here a short summary is provided with some notes to the features specific to open and wedged field verification.

For a given beam, the response of the amorphous silicon detectors is linear (D(Gy)=m*R+q). However response to primary or transmitted radiation is different and, in addition, dynamic deliveries like IMRT or EDW are changing dosimetric and geometrical conditions continuously during delivery. GLAaS accounts for those changes in time and position, using different *m *and *q *values, and differentiating between primary and transmitted (below the MLC or physical wedges) radiation, on a pixel by pixel basis.

The total dose *d*_*i *_in the *i-th *pixel, over the entire IMRT field delivery is:

(1) $$d_{i}=d_{pr,i}+d_{tr,i}=\left(\sum_{s=1}^{N}m_{pr,s}(EwwF)\cdot r_{i,s}+q_{pr,s}\right)+\left(m_{tr}\cdot \left(R_{i}-\sum_{s=1}^{N}r_{i,s}\right)+q_{tr}\right)$$

where: *m *and *q *are the slope and the intercept for a field of size *EwwF *(Equivalent window width Field), *r *is the reading attributed to the primary radiation for the beam ''segment" *s*, and *R *is the total PV reading; subscripts *pr *refer to primary, *tr *to transmitted radiation. The field is considered as a sum of *N *segments. For IMRT fields the definition of segments is straightforward (also in the case of dynamic sliding window). For open fields there is obviously only one segment as well as for hard wedged fields. For dynamic wedges, in principle it should be necessary to define a sequence of segments of progressively smaller size, following the jaws during motion. In practice it is sufficient to use one single segment, defined by the largest jaws opening since this contribution dominates over the entire field delivery. In these cases equation (1) becomes, inside the field:

(1a) $$d_{i}=d_{pr,i}=\left(\sum_{s=1}^{N}k\cdot m_{pr,s}(EwwF)\cdot r_{i,s}+q_{pr,s}\right)$$

While outside the field the only second term of eq. (1) is used.

The parameter values computed during the configuration of the GLAaS to analytically obtain the slopes come from the following empirical algorithm:

(2) *OF(EwwF) *= [*c *+ *d *· ln(*EwwF*)]^-1^

where *EwwF *is the equivalent field size of each segment

(3) *m*_*pr*_(*OF*) = *a *· *OF *+ *b*

where *m*_*pr *_is the slope for primary radiation, and *OF *is the PV measured output factor as per equation (2).

For transmitted radiation the following relationship is used:

(4) *m*_*tr *_= *k *· *m*_*pr*_

The parameter *k *depends on the type of field to measure. It is applied to radiation transmitted through the MLC, *k*~1.08. In the case of hard wedged fields, equation (1*), *k *ranges from 1.02 to 1.05 while for dynamic wedges, still according to eq. (1*), *k *= 1.

GLAaS configuration consists in the determination of a set of empirical parameters: *a*, *b*, *c*, *d*, *k*, *q*_*pr *_and *q*_*tr*_.

Experimental set-up: the measured PV images are acquired without adding any build-up on the top of the cassette. The measuring depth is considered to be 0.8 cm, i.e. the intrinsic water-equivalent thickness of the EPID device, but measurements are converted to dose at a depth equal to d_max_. Measurements are then compared with doses either measured with other detectors or computed by the TPS at d_max _in water. In this way the configuration relates PV acquisitions performed without adding any build-up material on the top of the PV cassette with doses calculated/measured at d_max_, where the definition of dose is more reliable. This procedure is similar to that usually applied when in-vivo dosimetry is performed with solid-state diodes without sufficient build-up material.
